# The Mincle-Activating Adjuvant TDB Induces MyD88-Dependent Th1 and Th17 Responses through IL-1R Signaling

**DOI:** 10.1371/journal.pone.0053531

**Published:** 2013-01-07

**Authors:** Christiane Desel, Kerstin Werninghaus, Manuel Ritter, Katrin Jozefowski, Jens Wenzel, Norman Russkamp, Ulrike Schleicher, Dennis Christensen, Stefan Wirtz, Carsten Kirschning, Else Marie Agger, Clarissa Prazeres da Costa, Roland Lang

**Affiliations:** 1 Institute of Clinical Microbiology, Immunology and Hygiene, University Hospital Erlangen, Erlangen, Germany; 2 Institute of Medical Microbiology, Immunology and Hygiene, Technische Universität München, Munich, Germany; 3 Statens Serum Institut, Department of Infectious Disease Immunology, Copenhagen, Denmark; 4 Medical Clinic 1, Gastroenterology, Pneumology and Endocrinology, University Hospital Erlangen, Erlangen, Germany; 5 Institut für Medizinische Mikrobiologie, Essen, Germany; Institut de Pharmacologie et de Biologie Structurale, France

## Abstract

Successful vaccination against intracellular pathogens requires the generation of cellular immune responses. Trehalose-6,6-dibehenate (TDB), the synthetic analog of the mycobacterial cord factor trehalose-6,6-dimycolate (TDM), is a potent adjuvant inducing strong Th1 and Th17 immune responses. We previously identified the C-type lectin Mincle as receptor for these glycolipids that triggers the FcRγ-Syk-Card9 pathway for APC activation and adjuvanticity. Interestingly, *in vivo* data revealed that the adjuvant effect was not solely Mincle-dependent but also required MyD88. Therefore, we dissected which MyD88-dependent pathways are essential for successful immunization with a tuberculosis subunit vaccine. We show here that antigen-specific Th1/Th17 immune responses required IL-1 receptor-mediated signals independent of IL-18 and IL-33-signaling. ASC-deficient mice had impaired IL-17 but intact IFNγ responses, indicating partial independence of TDB adjuvanticity from inflammasome activation. Our data suggest that the glycolipid adjuvant TDB triggers Mincle-dependent IL-1 production to induce MyD88-dependent Th1/Th17 responses *in vivo*.

## Introduction

Recombinant subunit vaccines are cheap and safe, but only weakly immunogenic unless adjuvants are used. The most commonly used human adjuvant Aluminium hydroxide (Alum) potently induces antibody responses but does not induce Th1 cellular immunity. Thus, new adjuvants are urgently needed to potentiate cell-mediated immune (CMI) responses crucial for protection against intracellular bacteria, e.g. *Mycobacterium tuberculosis*. A prerequisite for an efficient adjuvant is the activation of antigen presenting cells (APCs) by ligands for pattern recognition receptors. The choice of adjuvant(s) critically determines the type of memory response elicited, depending on the receptors and pathways triggered in APC *via* generation of cytokine milieus directing Th cell differentiation. E.g., the TLR9 ligand CpG ODN drives strong Th1 responses, whereas cationic dimethyldioctadecylammonium (DDA) liposomes containing the glycolipid trehalose-6,6-dibehenate (TDB), the synthetic analog of the mycobacterial cord factor trehalose-6,6-dimycolate, potently induce a strong Th17 in addition to Th1 immune response [1], [2]. DDA/TDB (also known as CAF01) is a next generation adjuvant and has entered clinical studies for vaccination with the recombinant *Mycobacterium tuberculosis* fusion protein Ag85B-ESAT-6 (H1) [3], [4]. We and others [5] identified the C-type lectin (CLR) Mincle as receptor for these glycolipids that triggers the FcRγ-Syk-Card9 pathway for APC activation and adjuvanticity [6]. *In vitro*, APC activation was solely dependent on recognition of TDB by Mincle, whilst MyD88 was dispensable. Surprisingly, TDB-immunized MyD88^−/−^ mice failed to mount antigen-specific Th1 immune responses [7]. Since this was unexpected and contradictory to our *in vitro* results, we investigated *in vivo* requirements of known MyD88-utilizing signaling events in immunization experiments using DDA/TDB and H1. Development of antigen-specific Th1 and Th17 immune responses was dependent on IL-1/IL-1R-mediated signals. Interestingly, inflammasome activation *via* ASC only partially accounted for CMI induction upon immunization with the glycolipid-containing adjuvant DDA/TDB.

## Results

### Mincle and MyD88 are Required for Induction of Th1 and Th17 Responses *in vivo*

**Figure 1:**
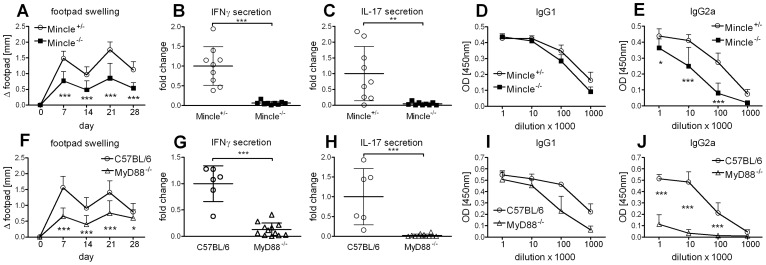
Mincle and MyD88 are required for DDA/TDB adjuvanticity. Footpad swelling (*A*), IFNγ, IL-17 (*B, C*) secretion and H1-specific antibodies (*D, E*) in Mincle^−/−^ and littermate controls. Data presented as mean ± SD pooled from 2 independent experiments with 4-5 mice/group. Footpad swelling (*F*), IFNγ, IL-17 (*G, H*) secretion and H1-specific antibodies (*I, J*) in MyD88^−/−^ and C57BL/6 controls. Data presented as mean ± SD pooled from 3 independent experiments with 2-5 mice/group. Cytokine production of cells isolated from the draining lymph nodes.

We have previously shown that the cationic liposome formulation DDA/TDB induces not only a strong Th1 immune response but also a Th17 response [2]. Vaccination with DDA/TDB induces stable, long-lived multifunctional CD4 memory T cells [8], [9]. We also found IFNγ and IL-17 secreted predominantly by CD4 T cells upon re-stimulation, while contribution of innate cells to IL-17 release was negligible (Fig. S1). This adjuvant effect depended on recognition of the synthetic glycolipid TDB by the CLR Mincle, when analyzed 7 days after a single subcutaneous (s.c.) immunization at the base of tail [6]. Here, we performed s.c. footpad prime-boost immunizations in order to utilize local swelling as an additional readout. Substantial swelling, peaking 7 days post immunization, was observed and significantly reduced in the absence of Mincle (Fig. 1*A*). This was in line with dramatically reduced secretion of IFNγ and IL-17 upon antigen-specific restimulation with H1 protein (Fig. 1*B, C*). Formation of H1-specific IgG2a antibodies, associated with Th1 responses, was also reduced whereas Th2 polarized IgG1 antibodies developed independently of Mincle (Fig. 1*D, E*). Footpad swelling was also significantly reduced in the absence of MyD88 (Fig. 1*F*), as well as IFNγ (Fig. 1*G*) and IL-17 (Fig. 1*H*) secretion. In addition, H1-specific IgG2a antibody generation was strongly impaired in the absence of MyD88, whereas the reduction of H1-specific IgG1 was not significant (Fig. 1*I, J*). Thus, in contrast to *in vitro* APC activation which solely required Mincle, *in vivo* adjuvanticity also strongly depends on MyD88 signaling.

**Figure 2:**
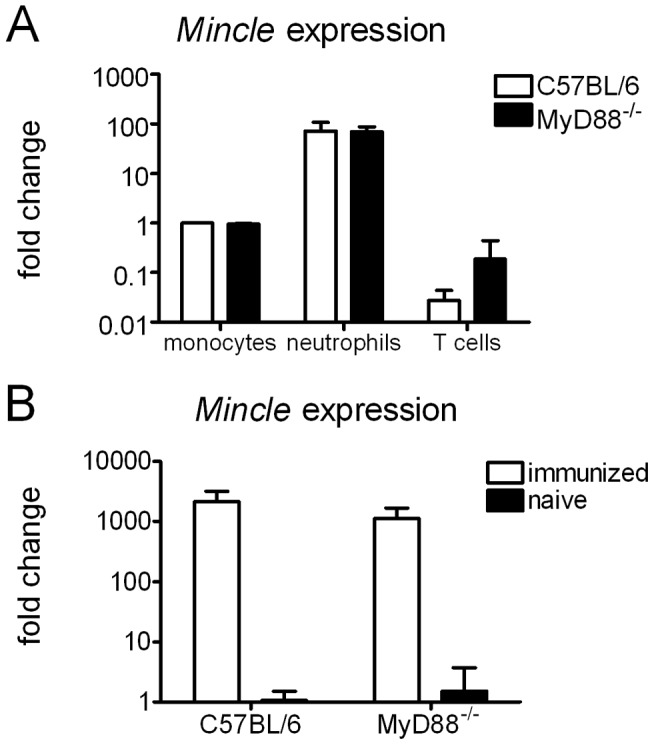
Basal and DDA/TDB-induced Mincle expression is not reduced in the absence of MyD88. Mincle expression determined by qRT-PCR in sorted cells from naive C57BL/6 or MyD88^−/−^ mice (*A*). Fold change calculated against sorted monocytes from C57BL/6 mice. Data presented as mean ± SD pooled from 2 independent sorts. Mincle expression determined by qRT-PCR in vaccinated and naïve mice (*B*). RNA isolated from footpads 3 days post immunization. Fold change calculated against naive C57BL/6. Data presented as mean ± SD pooled from 2 independent experiments with 2–3 mice/group.

Expression of Mincle by macrophages is inducible *in vitro* by TLR stimuli [10]. Therefore, it was possible that MyD88^−/−^ mice have reduced Mincle levels due to a lack of responsiveness to TLR signals, derived e.g. from the gut flora, which could account for abrogated inflammatory and immune responses observed in MyD88^−/−^ mice after immunization. To test this possibility, we first measured Mincle expression by qRT-PCR in FACS-sorted monocytes, neutrophils and T cells from naive C57BL/6 and MyD88-deficient mice (Fig. 2*A*). Expression was much higher in neutrophils than in monocytes, whereas T cells expressed very little Mincle mRNA. Of note, expression in monocytes and neutrophils was equally high in MyD88^−/−^ as in control cells. Expression of Mincle was also determined at the site of injection (Fig. 2*B*). In naive C57BL/6 and MyD88^−/−^ mice, Mincle mRNA was nearly undetectable but increased more than 3 orders of magnitude in both genotypes, most likely reflecting the infiltration by inflammatory leukocytes. Thus, absence of the adjuvant effect in MyD88^−/−^ mice cannot be attributed to a lack of Mincle expression.

Since MyD88 is the common adaptor molecule in TLR-mediated signaling, we analyzed TLR2,3,4,7 quadruple knockout and TLR9^−/−^, all of which responded normally to immunization with TDB (Fig. S2). Since TLR2 pairs with TLR1 and TLR6, these experiments addressed seven of the eleven murine TLRs. As TLR5 binds flagellin, TLR8 is non-functional in mice, and TLR11 is the receptor for toxoplasma profilin, we consider it unlikely, that TLR-dependent signaling is required for DDA/TDB adjuvanticity *in vivo*. However, we cannot formally exclude that one or more of these TLRs contribute to the DDA/TDB effect.

### IL-1 Receptor Signaling Mediates Th1 and Th17 Cell Induction

**Figure 3:**
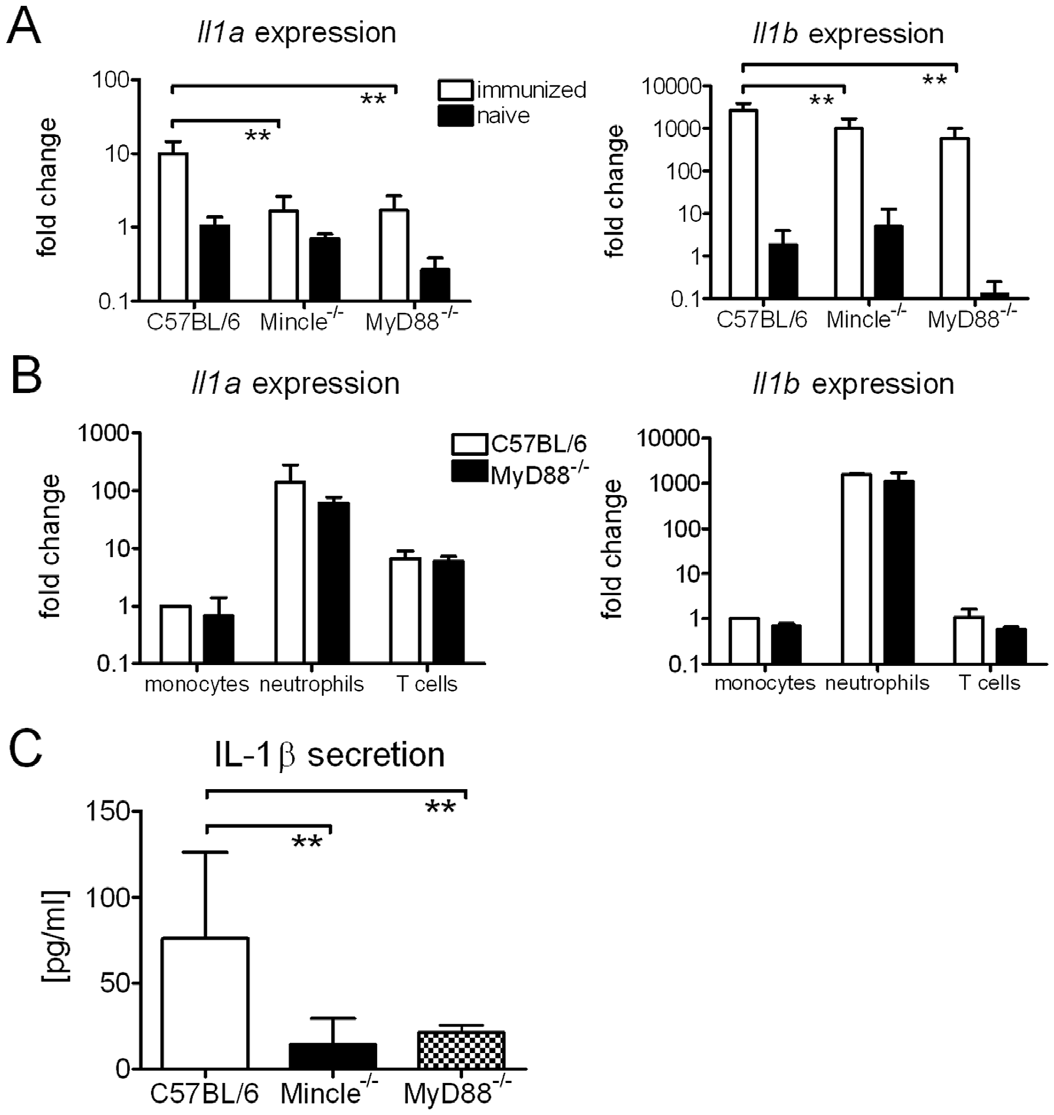
TDB induces IL-1 expression and release *in vitro* and *in vivo*. *Il1a* and *Il1b* expression determined by qRT-PCR in vaccinated and naive mice (*A*). RNA isolated from footpads 3 days post immunization. Fold change calculated against naive C57BL/6. Data presented as mean ± SD pooled from 2 independent experiments with 2–3 mice/group. Expression of *Il1a* and *Il1b* in sorted immune cells from naive C57BL/6 and MyD88^−/−^ mice (*B*). Fold change calculated against sorted monocytes from C57BL/6 mice. Data presented as mean and SD pooled from 2 independent sorts. IL-1β release of 5×10^5^ cells isolated from the footpad 7 days post immunization, cultured over night in medium (*C*). Data presented as mean ± SD pooled from 3 independent experiments with 3 mice/group.

MyD88 also conveys signaling of the receptors for IL-1, IL-18 and IL-33 [11]–[14]. TDB induces the expression and secretion of IL-1β from macrophages *in vitro* [2]. We therefore analyzed IL-1 expression at the site of injection and detected increased mRNA levels of *Il1b* and to a lesser extent also *Il1a* in the feet 3 days post immunization (Fig. 3*A* *)*. These changes at the mRNA level were at least in part due to leukocyte infiltration, as neutrophils constitutively express *Il1a* and *Il1b* (Fig. 3*B*). Importantly, cells isolated from footpads 7 days post immunization secreted IL-1β after over night incubation in culture medium (Fig. 3*C*). This was strongly dependent on Mincle and MyD88.

**Figure 4:**
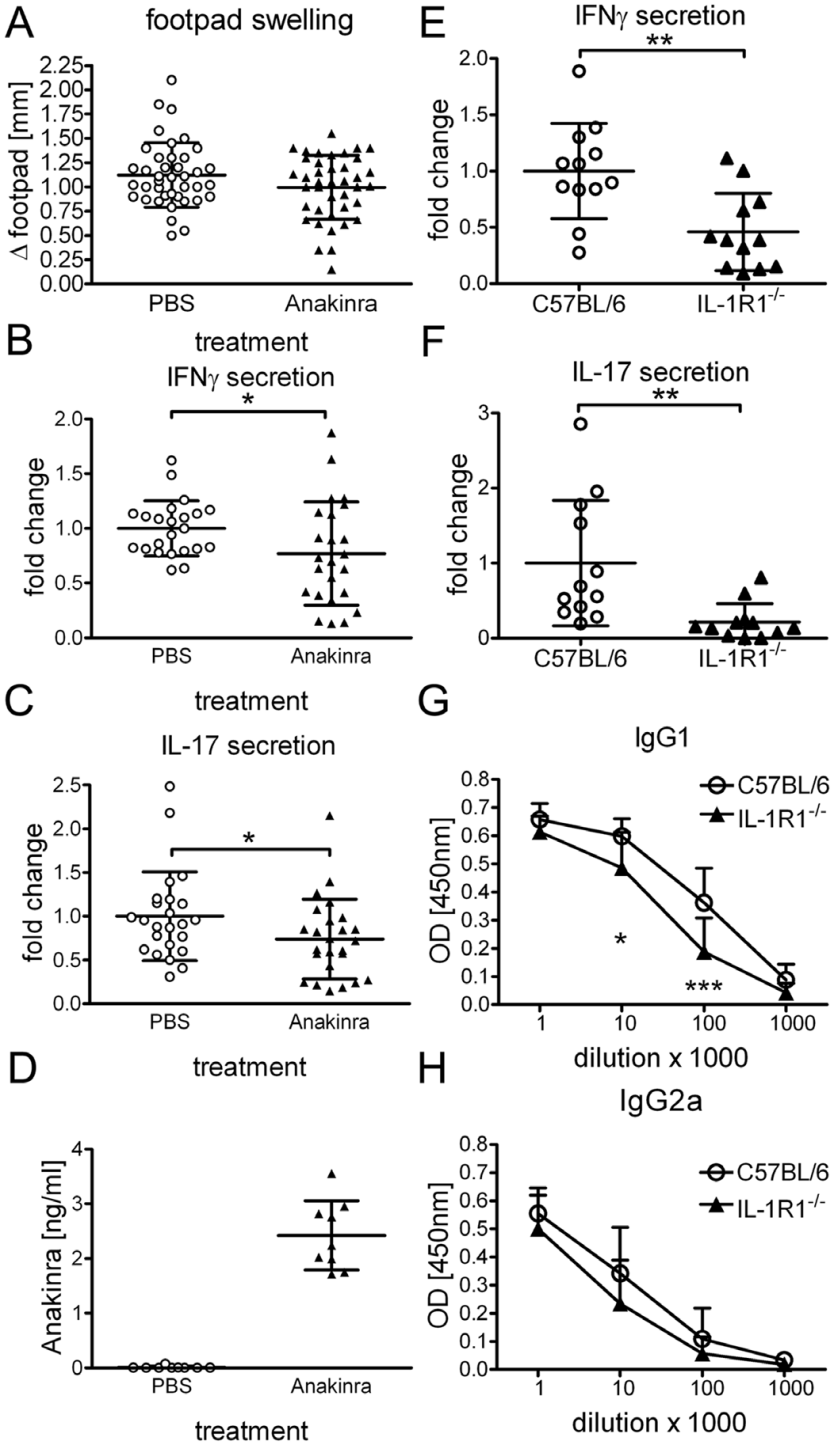
IL-1R1 signaling contributes to DDA/TDB adjuvanticity. Footpad swelling (*A*), IFNγ, IL-17 (*B, C*) secretion of cells isolated from the draining lymph nodes and Anakinra serum concentration (*D*) 7 days post immunization. Daily injection of 100 µg/g Anakinra or corresponding volume of PBS i.p.; first treatment 3–6 hours prior to immunization. Data presented as mean ± SD pooled from 5 independent experiments with 4–5 mice/group; except (*D*). IFNγ, IL-17 secretion of splenocytes (*E, F*) and H1-specific antibodies (*G, H*) in IL-1R1^−/−^ and C57BL/6 controls; base of tail immunization. Data presented as mean ± SD pooled from 2 independent experiments with 5–6 mice/group.

To assess the contribution of IL-1 receptor signals to adjuvanticity of TDB, we next investigated whether blockade of IL-1R with the soluble IL-1 receptor antagonist Anakinra influences the outcome of immunization. Anakinra treatment did not affect footpad swelling (Fig. 4*A*), but significantly reduced IFNγ and IL-17 secretion (Fig. 4*B, C*). Even though the serum concentration of Anakinra was consistently high in all mice treated (Fig. 4*D*), we do not know whether Anakinra treatment completely inhibited IL-1-mediated signaling. Therefore, we immunized IL-1R1^−/−^ mice with DDA/TDB/H1. Due to differences in animal protection regulations in the different animal facilities, IL-1R1^−/−^ mice were vaccinated s.c. at the tail base. Requirement for IL-1-mediated signals was more pronounced in these experiments as compared to short term Anakinra treatment. Cytokine secretion was significantly reduced (Fig. 4*E, F*); albeit not as strongly as seen in MyD88^−/−^ mice. Interestingly, even though antibody generation was dependent on MyD88 signaling, absence of IL-1R1 did not alter IgG2a and only marginally affected IgG1 antibody formation (Fig. 4*G, H*). Thus, IL-1/IL-1R1 signaling contributes to the development of a Th1/Th17 immune response upon immunization with a CLR triggering adjuvant without strong effects on B cell responses or isotype switching.

### IL-18 and IL-33 are Dispensable for Adjuvanticity

**Figure 5:**
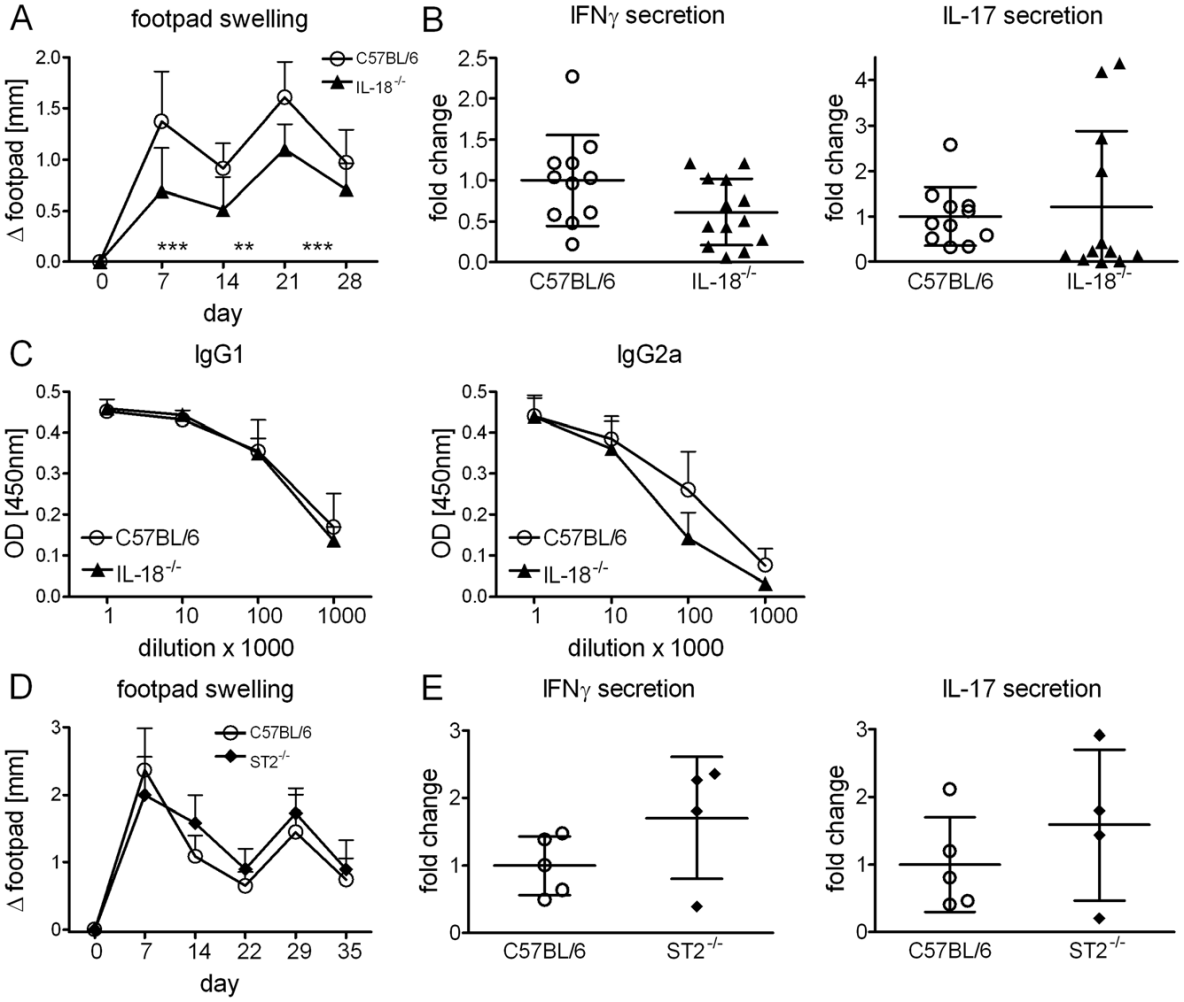
IL-18 and IL-33 are dispensable for DDA/TDB adjuvanticity. Footpad swelling (*A*), IFNγ, IL-17 secretion of cells isolated from the draining lymph nodes (*B*) and H1-specific antibodies (*C*) in IL-18^−/−^ and C57BL/6 controls. Data presented as mean ± SD pooled from 3 independent experiments with 3–5 mice/group. Footpad swelling (*D*), IFNγ and IL-17 secretion of cells isolated from the draining lymph nodes in ST2^−/−^ and C57BL/6 controls (*E*). Immunization at day 0 and day 21, mice sacrificed at day 35. Data presented as mean ± SD from 1 experiment with 4–5 mice/group.

We next immunized IL-18^−/−^ mice. Footpad swelling was significantly reduced (Fig. 5*A*), however IL-18 signaling was not required for Th1/Th17 response induction (Fig. 5*B*). Development of humoral immune responses remained also unchanged in the absence of IL-18 (Fig. 5*C*). Thus, contribution of IL-18/IL-18R signaling was rather negligible although it does mediate local inflammation at the injection site. Finally, in mice deficient in the IL-33 receptor component ST2, footpad swelling and secretion of IFNγ and IL-17 were not impaired after immunization (Fig. 5*D, E*).

### Canonical Inflammasome Activation Contributes to Th17 but not Th1 Responses

IL-1β and IL-18 have to be cleaved in order to exert their proinflammatory effects. This is mediated by caspase-1 in a process called inflammasome activation [15], [16]. Since IL-1-mediated signaling contributed to DDA/TDB adjuvanticity, we asked whether inflammasome activation was also a requirement for the adjuvant effect. An essential adapter protein for inflammasome activation is ASC. To address the control of IL-1 production in response to the glycolipid adjuvant, we first stimulated bone marrow-derived DC from ASC^−/−^ as well as Mincle^−/−^ and MyD88^−/−^ mice *in vitro*. Stimulation of DC with TDB induced expression of *Il1a, Il1b* and of *Csf3* (encoding G-CSF) with complete dependence on Mincle, but independent of MyD88 and ASC (Fig. S3 *A*). At the protein level, IL-1β was detected in the supernatant in a Mincle- and ASC-dependent, albeit MyD88-independent manner, whereas IL-1α protein was absent in Mincle^−/−^ DC cultures, but independent of ASC (Fig. S3 *B*). Of note, while *Il1a* and *Il1b* mRNA induction was triggered by immobilized TDB as well as by TDB in suspension, both IL-1 proteins where secreted only when DC were stimulated with the particulate TDB in suspension (Figure S3 *A, B*), indicating that phagocytosis of glycolipids is a prerequisite for ASC-dependent release of IL-1β as well as the ASC-independent secretion of IL-1α.

ASC was shown to fulfil an important inflammasome-independent function in post-transcriptional regulation of cytoskeletal rearrangements and lack of ASC impaired antigen uptake and priming capacity of bone marrow-derived DC as well as chemotaxis of lymphocytes; all due to absent Dock2-signals [17]. Recently, the same group reported that not all of the different ASC^−/−^ mouse lines available show defective Dock2 expression [18]; likely resulting in differences regarding the antigen presentation and migratory capacity of lymphoid and myeloid cells. We have used the ASC^−/−^ mice originally generated at Genentech [19] and detected comparable Dock2 expression by qRT-PCR (data not shown). We tested ASC^−/−^ bone marrow-derived macrophages in phagocytosis and spreading assays and found both unimpaired (Figure S4), arguing against an inflammasome-independent requirement for ASC in TDB-mediated adjuvanticity.

**Figure 6:**
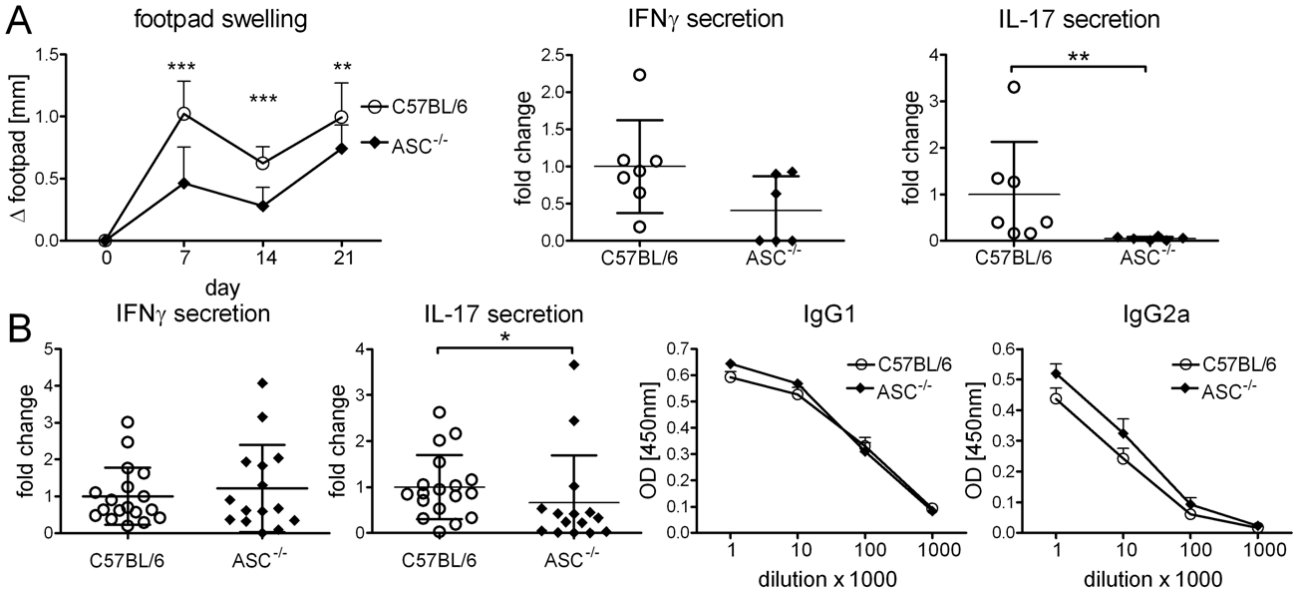
Partial contribution of ASC to DDA/TDB adjuvanticity. Footpad swelling, IFNγ and IL-17 secretion in ASC^−/−^ and C57BL/6 controls (*A*). Data presented as mean ± SD pooled from 2 independent experiments with 3–5 mice/group. IFNγ, IL-17 secretion and H1-specific antibodies in ASC^−/−^ and C57BL/6 controls; base of tail immunization (*B*). Data presented as mean ± SD pooled from 3 independent experiments with 5–6 mice/group. Re-stimulation of cells isolated from the draining lymph nodes.

We next investigated the impact of ASC-deficiency on adjuvanticity *in vivo* (Fig. 6). Following footpad immunization of ASC^−/−^ mice, local swelling and induction of IL-17 secreting cells was significantly reduced, whereas IFNγ induction was unaltered (Fig. 6*A*). In a set of experiments employing base of tail immunization, a significant effect of ASC-deficiency was again observed for IL-17 induction but not for IFNγ secretion (Fig. 6*B*). Antibody responses were not altered in ASC^−/−^ mice. Thus, the lack of ASC has less severe consequences for induction of Th responses by DDA/TDB than the loss of IL-1R signaling, indicating that inflammasome activation contributes to but does not completely account for generation of IL-1-dependent signaling through MyD88 *in vivo*.

## Discussion

Here we show that CMI induction upon immunization with the cationic adjuvant formulation DDA/TDB essentially requires MyD88 signaling. Our evidence for a TLR-independent role of MyD88 signaling in response to TDB stimulation is in line with data from Geisel et al. showing that cell recruitment and cytokine production induced by trehalose dimycolate was severely reduced in MyD88^−/−^ mice but unaffected in TLR2^−/−^ and TLR4^−/−^ mice [20]. The strong MyD88-dependence observed here contrasts to the results obtained by Gavin et al. claiming that MyD88 is dispensable for generation of T cell dependent antibody responses upon vaccination with a range of adjuvants [21]. This discrepancy may be due to differences in the antigen (whole protein vs. hapten antigen) or the readout used (Th cell polarization vs. antibody responses).

We identified here IL-1/IL-1R1 as the TLR-independent MyD88 pathway significantly contributing to induction of Th1 and Th17 cellular immune responses upon vaccination with DDA/TDB. IL-1 has been linked to induction of pathogenic Th17 cells in an EAE model [22] but was also shown to induce protective Th17 immune responses using *Escherichia coli* heat-labile enterotoxin as adjuvant [23]. Furthermore IL-1β and IL-18 can promote IL-17 and IFNγ production in CD4 and γδ T cells [24], in line with our data showing that both Th1 and Th17 induction is reduced in the absence of IL-1R1. Of note, CMI generation was reduced in IL-1R1^−/−^ mice to a lesser extent than in MyD88^−/−^ mice, raising the question which other factors explain the strong MyD88-dependence. One possible explanation could be synergistic effects of IL-1 and IL-18, which promotes Th1 T cell differentiation together with IL-12 [25]. Lalor et al. also suggest redundancy for IL-1β and IL-18 in Th17 induction [24]. We did not detect reduced CMI induction in IL-18^−/−^ mice where IL-1/IL-1R1 is still functional; however, local inflammation in the footpad was reduced in IL-18^−/−^ as in MyD88^−/−^ mice. Although IL-1R1 signaling was required for robust IFNγ production by T cells, the Th1-associated IgG2a antibody response was unimpaired in IL-1R1^−/−^ mice (Fig. 4). The basis for IL-1R1-independent, MyD88-dependent antigen-specific B cell responses is currently unkown; in future experiments, we plan to use conditional MyD88 transgenic mice to investigate the requirement for MyD88 in T and B lymphocytes, as well as myeloid cells, for efficient isotype switching.

In addition, the inducibility of Mincle by TLR ligands [10] raised the possibility that MyD88^−/−^ mice may be less responsive to TDB as adjuvant because of reduced receptor expression. However, our results showing that Mincle expression is also increased in MyD88^−/−^ mice upon immunization and that basal expression of Mincle in sorted monocytes, neutrophils and T cells is comparable between MyD88^−/−^ and C57BL/6 control mice (Fig. 2) allow us to discard this possibility. Instead, impaired responses to other cytokines and chemokines, e.g. IFNγ [26] might contribute to the observed lack of responsiveness.

Unexpectedly, the adjuvant effect of DDA/TDB, even though dependent on IL-1/IL-1R1, was less strongly reliant on the inflammasome adaptor molecule ASC. Here again, DDA/TDB differed in the requirements for induction of immune responses compared to other known adjuvants, i.e. Alum which induces Th2 responses *via* Nlrp3-mediated inflammasome activation [27]–[29]. Schweneker et al. recently showed that TDB activates the Nlrp3 inflammasome in an ASC-dependent manner [30]. IL-1β secretion from bone marrow-derived DC upon stimulation with TDB was dependent on Caspase-1, Nlrp3 and ASC. It was further demonstrated that phagocytosis of TDB was a prerequisite for inflammasome activation. This is in line with our data (Fig. S3) showing also Mincle- and ASC-dependent IL-1β secretion only upon stimulation with TDB in suspension, whereas *Il1b* expression was increased independent of uptake and inflammasome activation. However, in the context of vaccination with DDA/TDB, dependency on ASC was less pronounced as compared to the loss of IL-1R signaling. One possible explanation could be that neutrophils utilize serine proteases to cleave pro-IL-1β [31]. Massive neutrophil influx is a hallmark of DDA/TDB adjuvanticity (data not shown) and we detected very high expression of *Il1b* in sorted neutrophils isolated from naive C57BL/6 and MyD88^−/−^ mice (Fig. 3*B*). Alternatively, ASC-independent non-canonical inflammasome activation *via* Caspase-11 and subsequent IL-1α release [32] may be operating. IL-1α, in contrast to IL-1β, is active as precursor as well as the calpain-cleaved mature form [33]. Gross et al. recently reported that particulate activators of Nlrp3 and strong inducers of calcium influx induced processing and secretion of IL-1α in an inflammasome-independent manner [34]. We also detected high expression of *Il1a* in sorted neutrophils (Fig. 3*B*) isolated from naive C57BL/6 and MyD88^−/−^ mice. Bone marrow-derived DC secreted IL-1α upon stimulation with TDB in suspension in a Mincle- and MyD88-dependent, yet ASC-independent manner (Fig. S3 *B*). As IL-1α and IL-1β both bind to the IL-1R and are antagonized by Anakinra, ASC-independent release of IL-1α may make an important contribution to DDA/TDB-induced Th induction.

Our results suggest the following scenario of induction and effects of IL-1 following TDB-adjuvanted immunization: 1. Recognition of TDB by Mincle triggers transcriptional upregulation of *Il1a* and *Il1b* mRNA, independent of MyD88 and ASC *via* Syk-Card9. 2. Uptake of TDB by DC (and possibly other myeloid cells) activates the Nlrp3 inflammasome and triggers IL-1β secretion in an ASC-dependent manner, whereas IL-1α is released independent of ASC. 3. Both IL-1 proteins trigger IL-1R signaling via MyD88 to amplify inflammation at the site of injection and to direct Th differentiation to Th17 [35] and Th1 cells.

Taken together, we have shown here that the glycolipid adjuvant TDB relies on MyD88-dependent pathways for efficient Th1/Th17 adjuvanticity. Pharmacologic and genetic abrogation of IL-1R signaling identified IL-1 as the major MyD88-dependent factor induced by TDB through Mincle-Card9 signaling, providing new insight into the adjuvant mechanism. As DDA/TDB has entered clinical trials, further dissection of IL-1R-dependent effects on innate and adaptive immune cells will be relevant for a detailed understanding of the molecular mode of action of this adjuvant.

## Materials and Methods

### Ethics Statement

All procedures were discussed with and approved by the animal protection committees of regional Bavarian governments (Regierung von Mittelfranken or Oberbayern animal protocols number 54-2532.1.12/09 and 211-2531-33/05) according to German animal protection law (BGBI.I S. 1206, 1313).

### Mice

MyD88^−/−^ mice were used with permission of Dr. S. Akira [11], Mincle^−/−^ mice have been described [36]. IL-1R1^−/−^ and ASC^−/−^ mice were bred at the Technische Universität München. ASC^−/−^ were originally generated at Genentech [19]. C57BL/6, Mincle^−/−^, MyD88^−/−^, ST2^−/−^, IL-18^−/−^ and TLR9^−/−^ mice were bred at the animal facility of the Medical Faculty in Erlangen. For some experiments, C57BL/6 mice were purchased from Harlan or Charles River.

### Immunization and IL-1 Receptor Blockade

DDA/TDB liposomes and recombinant H1 were provided by the Statens Serum Institut. Adjuvant formulations were prepared as described [2]. Mice were immunized twice in a 14-day interval unless otherwise stated. IL-1 receptor signaling was blocked by intraperitoneal injection of 100 µg/g body weight soluble IL-1 receptor antagonist Anakinra (Kineret, Amgen) daily. Since Anakinra had to be given daily and previous experiments showed IFNγ and IL-17 production could be detected 7 days after a single immunization [6], we chose a 7-day immunization protocol. Anakinra serum concentration was determined by sandwich ELISA using anti-human IL-1-RA antibodies (BioLegend).

### Antigen-specific Restimulation and Detection of H1-specific Antibodies

Mice were sacrificed two weeks after the second immunization unless stated otherwise. Pooled draining inguinal and popliteal lymph nodes or spleens were meshed through a 100 µm nylon sieve and 5×10^5^ cells were restimulated with 10 µg/ml H1 protein for 96 hours. Supernatants were analyzed for IFNγ and IL-17 production by ELISA (R&D Systems). Background (unstimulated cells) was subtracted and cytokine release expressed as fold change relative to the mean response of restimulated cells from immunized control mice. We chose to present summarized data as fold change in order to account for inter-experimental variation in overall cytokine production because experiments were conducted in different laboratories and with mice from different animal houses. Serum was analyzed in tenfold dilutions (starting from 1/1,000) for H1-specific antibodies with rabbit anti-mouse IgG1 and IgG2a (BD Biosciences). Pooled serum from immunized mice was included on each ELISA plate as a positive inter-assay control.

### Intracellular Cytokine Staining

2×10^6^ cells isolated from draining lymph nodes 7 days after the second immunization were re-stimulated with a mixture of 10 µg/ml H1 protein and 5 µg/ml of the peptides: Ag85B CD8 epitope (p1–19): FSRPGLPVEYLQVPSPSMG, Ag85B CD4 epitope (p241–255): QDAYNA-AGGHNAVFN, Ag85B CD4 epitope (p261–280): THSWEYWGAQLNAMKGDLQS and ESAT-6 (p1–15): MTEQQWNFAGIEAAA. After 1 h 10 µg/ml Brefeldin A was added and incubation continued for 23 h. PMA (50 ng/ml)/ionomycin (1 µg/ml) was used as a positive and culture medium as negative controls. Intracellular cytokine staining was performed according to standard protocols with antibodies against CD3, CD4, CD8, NK1.1, γδ TCR, CD11b, IFNγ and IL-17 (eBioscience or BioLegend) and data recorded on a FACSCanto II (BD Biosciences).

### Generation and Stimulation of Bone Marrow-derived DC

Bone marrow cells were cultured on Petri dishes for 8 days in cRPMI containing 10% X63-cell conditioned medium. 2.5×10^5^ cells/ml (cytokine secretion) or 5×10^5^ cells/ml (qRT-PCR) were stimulated as indicated with plate coated TDB [2] or TDB in suspension (TDB suspended in DMEM by vortexing, heating to 60°C and 10 min sonication). IL-1α and IL-1β release was determined by ELISA (eBioscience).

### Single Cell Isolation from Footpad

The injection site was excised using a scalpel and snap frozen in liquid nitrogen for qRT-PCR analysis. RNA was isolated using TriFast (peqlab) according to the manufacturer’s protocol. For IL-1β release single cell suspensions were obtained using gentleMACS (Miltenyi Biotec) according to the manufacturer’s protocol and 5×10^5^ cells cultured in 200 µl cDMEM over night.

### Quantitative RT-PCR

Expression levels of the housekeeping gene *Hprt* as well as *Il1a*, *Il1b*, *Csf3* and *Mincle* were analyzed using primer/probe combinations selected from the Roche Universal Probe Library. Fold changes were calculated with the ΔΔCT method using calibrators as indicated.

### Cell Sorting

Single cell suspensions from bone marrow and spleen were stained with antibodies against CD11b, Ly6C, Ly6G (bone marrow) or CD3, CD19 (spleen); all antibodies from eBioscience. Monocytes (CD11b^+^Ly6C^+^Ly6G^−^), neutrophils (CD11b^+^Ly6C^+^Ly6G^+^) and T cells (CD3^+^CD19^−^) were sorted on a MoFlo (Beckman Coulter). 5×10^5^−1×10^6^ sorted cells were lysed in TriFast for RNA isolation and qRT-PCR.

### Phagocytosis and Spreading Assay

Spreading assay of bone marrow-derived macrophages (BMM) was performed as described [37]. For phagocytosis assays BMM were incubated with green fluorescent latex beads (1 µm; Flouresbrite Microparticles, Polyscience, Inc.) with an MOI of 20 and incubated for 1 h or 4 h at 37°C. Cells were washed twice with ice cold PBS, detached from the 24 well plate by using a cell scraper and resuspended in PBS containing 0,2% BSA. Percentages of living cells which had phagocytosed beads were determined using a FACSCanto II (BD Biosciences).

### Statistical Analysis

Statistical analysis was performed using Prism 5 from GraphPad Software, Inc. Significance was determined by 2-way ANOVA with Bonferroni correction for footpad swelling and H1-specific antibodies. For cytokine secretion normal distribution was tested by Shapiro-Wilk followed by unpaired student’s t-test for Gaussian or Mann-Whitney for non-Gaussian distribution. *p<0.05, **p<0.01 and ***p<0.001.

## Supporting Information

Figure S1 **IFNγ and IL-17 are secreted by** ***bona fide*** **Th1 and Th17 cells.** Number of IFNγ (*A*) and IL-17 (*B*) secreting cells determined by intracellular cytokine staining. Cells isolated from draining popliteal lymph nodes 7 days after the second immunization (footpad). 2×10^6^ cells per well, 24 h incubation with 10 µg/ml H1, PMA/Ionomycin or medium control in the presence of Brefeldin A. IFNγ (*C*) and IL-17 (*D*) release of the same cells (5×10^5^) stimulated for 96 h without Brefeldin A. One of two representative experiments with 2 mice/group shown.
(TIF)

Figure S2 **TLR2,3,4,7 and 9 seem dispensable for DDA/TDB adjuvanticity.** Footpad swelling, IFNγ and IL-17 secretion in TLR2,3,4,7^−/−^ and C57BL/6 controls (*A*). Data presented as mean ± SD from 1 experiment with 3 mice/group. Footpad swelling, IFNγ and IL-17 secretion in TLR9^−/−^ and C57BL/6 controls (*B*). Data presented as mean ± SD from 3 independent experiments with 3–5 mice/group. Cytokine production of cells isolated from the draining lymph nodes.
(TIF)

Figure S3 **TDB-induced expression and release of IL-1α and IL-1β.** Expression of *Il1a*, *Il1b* and *Csf3* (*A*), and IL-1α and IL-1β secretion (*B*). Bone marrow-derived DC were stimulated for 24 h with plate-coated TDB (solvent control isopropanol) or TDB in suspension. 2.5×10^5^ cells/well were seeded for cytokine release (TDB concentration as indicated) and 5×10^5^ cells/well for qRT-PCR (5 µg/ml TDB). Fold change calculated against DC from C57BL/6 mice in medium. One experiment performed in duplicates.
(TIF)

Figure S4 **ASC^−/−^ macrophages show no defects in spreading kinetics and phagocytosis capacity.** Spreading kinetics (*A*) of C57BL/6 (circles) and ASC^−/−^ (squares) BMM stimulated with LPS (closed symbols) or media control (open symbols). Mean ± SEM of at least 300 cells per condition. Statistical significance refers to the comparison of LPS stimulated C57BL/6 and ASC^−/−^ BMM. One experiment performed. Phagocytosis capacity of C57BL/6 (*B*) and ASC^−/−^ (*C*) BMM. Cells were incubated with fluorescent latex beads (1 µm; MOI 20) for 1 h and 4 h. Percentages of cells which phagocytosed beads determined flow cytometry. One of two representative experiments shown.
(TIF)

## References

[1] Davidsen J, Rosenkrands I, Christensen D, Vangala A, Kirby D, et al (2005) Characterization of cationic liposomes based on dimethyldioctadecylammonium and synthetic cord factor from *M. tuberculosis* (trehalose 6,6′-dibehenate)-a novel adjuvant inducing both strong CMI and antibody responses. Biochim Biophys Acta 1718: 22–31. 16321607 10.1016/j.bbamem.2005.10.011

[2] Werninghaus K, Babiak A, Gross O, Holscher C, Dietrich H, et al (2009) Adjuvanticity of a synthetic cord factor analogue for subunit *mycobacterium tuberculosis* vaccination requires FcRγ-syk-card9-dependent innate immune activation. J Exp Med 206: 89–97. 19139169 10.1084/jem.20081445 PMC2626670

[3] Kaufmann SH, Hussey G, Lambert PH (2010) New vaccines for tuberculosis. Lancet 375: 2110–2119. 20488515 10.1016/S0140-6736(10)60393-5

[4] Ottenhoff TH, Doherty TM, van Dissel JT, Bang P, Lingnau K, et al (2010) First in humans: A new molecularly defined vaccine shows excellent safety and strong induction of long-lived *mycobacterium tuberculosis*-specific TH1-cell like responses. Hum Vaccin 6: 1007–1015. 21178394 10.4161/hv.6.12.13143

[5] Ishikawa E, Ishikawa T, Morita YS, Toyonaga K, Yamada H, et al (2009) Direct recognition of the mycobacterial glycolipid, trehalose dimycolate, by C-type lectin mincle. J Exp Med 206: 2879–2888. 20008526 10.1084/jem.20091750 PMC2806462

[6] Schoenen H, Bodendorfer B, Hitchens K, Manzanero S, Werninghaus K, et al (2010) Cutting edge: Mincle is essential for recognition and adjuvanticity of the mycobacterial cord factor and its synthetic analog trehalose-dibehenate. J Immunol 184: 2756–2760. 20164423 10.4049/jimmunol.0904013 PMC3442336

[7] Agger EM, Rosenkrands I, Hansen J, Brahimi K, Vandahl BS, et al (2008) Cationic liposomes formulated with synthetic mycobacterial cordfactor (CAF01): A versatile adjuvant for vaccines with different immunological requirements. PLoS One 3: e3116. 18776936 10.1371/journal.pone.0003116 PMC2525815

[8] Lindenstrom T, Agger EM, Korsholm KS, Darrah PA, Aagaard C, et al (2009) Tuberculosis subunit vaccination provides long-term protective immunity characterized by multifunctional CD4 memory T cells. J Immunol 182: 8047–8055. 19494330 10.4049/jimmunol.0801592

[9] Lindenstrom T, Woodworth J, Dietrich J, Aagaard C, Andersen P, et al (2012) Vaccine-induced Th17 cells are maintained long-term postvaccination as a distinct and phenotypically stable memory subset. Infection & Immunity 80: 3533–3544. 22851756 10.1128/IAI.00550-12 PMC3457559

[10] Matsumoto M, Tanaka T, Kaisho T, Sanjo H, Copeland NG, et al (1999) A novel LPS-inducible C-type lectin is a transcriptional target of NF-IL6 in macrophages. J Immunol 163: 5039–5048. 10528209

[11] Adachi O, Kawai T, Takeda K, Matsumoto M, Tsutsui H, et al (1998) Targeted disruption of the myd88 gene results in loss of IL-1- and IL-18-mediated function. Immunity 9: 143–150. 9697844 10.1016/s1074-7613(00)80596-8

[12] Muzio M, Ni J, Feng P, Dixit VM (1997) IRAK (pelle) family member IRAK-2 and MyD88 as proximal mediators of IL-1 signaling. Science 278: 1612–1615. 9374458 10.1126/science.278.5343.1612

[13] Schmitz J, Owyang A, Oldham E, Song Y, Murphy E, et al (2005) IL-33, an interleukin-1-like cytokine that signals via the IL-1 receptor-related protein ST2 and induces T helper type 2-associated cytokines. Immunity 23: 479–490. 16286016 10.1016/j.immuni.2005.09.015

[14] Wesche H, Henzel WJ, Shillinglaw W, Li S, Cao Z (1997) MyD88: An adapter that recruits IRAK to the IL-1 receptor complex. Immunity 7: 837–847. 9430229 10.1016/s1074-7613(00)80402-1

[15] Ghayur T, Banerjee S, Hugunin M, Butler D, Herzog L, et al (1997) Caspase-1 processes IFN-gamma-inducing factor and regulates LPS-induced IFN-gamma production. Nature 386: 619–623. 9121587 10.1038/386619a0

[16] Martinon F, Burns K, Tschopp J (2002) The inflammasome: A molecular platform triggering activation of inflammatory caspases and processing of proIL-1beta. Mol Cell 10: 417–426. 12191486 10.1016/s1097-2765(02)00599-3

[17] Ippagunta SK, Malireddi RK, Shaw PJ, Neale GA, Walle LV, et al (2011) The inflammasome adaptor ASC regulates the function of adaptive immune cells by controlling Dock2-mediated rac activation and actin polymerization. Nat Immunol 12: 1010–1016. 21892172 10.1038/ni.2095 PMC3178750

[18] Ippagunta SK, Malireddi RK, Shaw PJ, Neale GA, Walle LV, et al (2012) Addendum: Defective Dock2 expression in a subset of ASC-deficient mouse lines. Nat Immunol 13: 701–702. 22905357 10.1038/ni.2389

[19] Mariathasan S, Newton K, Monack DM, Vucic D, French DM, et al (2004) Differential activation of the inflammasome by caspase-1 adaptors ASC and IPAF. Nature 430: 213–218. 15190255 10.1038/nature02664

[20] Geisel RE, Sakamoto K, Russell DG, Rhoades ER (2005) In vivo activity of released cell wall lipids of *mycobacterium bovis* bacillus calmette-guerin is due principally to trehalose mycolates. J Immunol 174: 5007–5015. 15814731 10.4049/jimmunol.174.8.5007

[21] Gavin AL, Hoebe K, Duong B, Ota T, Martin C, et al (2006) Adjuvant-enhanced antibody responses in the absence of toll-like receptor signaling. Science 314: 1936–1938. 17185603 10.1126/science.1135299 PMC1868398

[22] Sutton C, Brereton C, Keogh B, Mills KH, Lavelle EC (2006) A crucial role for interleukin (IL)-1 in the induction of IL-17-producing T cells that mediate autoimmune encephalomyelitis. J Exp Med 203: 1685–1691. 16818675 10.1084/jem.20060285 PMC2118338

[23] Brereton CF, Sutton CE, Ross PJ, Iwakura Y, Pizza M, et al (2011) *Escherichia coli* heat-labile enterotoxin promotes protective Th17 responses against infection by driving innate IL-1 and IL-23 production. J Immunol 186: 5896–5906. 21490151 10.4049/jimmunol.1003789

[24] Lalor SJ, Dungan LS, Sutton CE, Basdeo SA, Fletcher JM, et al (2011) Caspase-1-processed cytokines IL-1β and IL-18 promote IL-17 production by gamma delta and CD4 T cells that mediate autoimmunity. J Immunol 186: 5738–5748. 21471445 10.4049/jimmunol.1003597

[25] Okamura H, Tsutsi H, Komatsu T, Yutsudo M, Hakura A, et al (1995) Cloning of a new cytokine that induces IFN-gamma production by T cells. Nature 378: 88–91. 7477296 10.1038/378088a0

[26] Sun D, Ding A (2006) MyD88-mediated stabilization of interferon-gamma-induced cytokine and chemokine mRNA. Nat Immunol 7: 375–381. 16491077 10.1038/ni1308

[27] Eisenbarth SC, Colegio OR, O’Connor W, Sutterwala FS, Flavell RA (2008) Crucial role for the Nalp3 inflammasome in the immunostimulatory properties of aluminium adjuvants. Nature 453: 1122–1126. 18496530 10.1038/nature06939 PMC4804622

[28] Kool M, Soullie T, van Nimwegen M, Willart MA, Muskens F, et al (2008) Alum adjuvant boosts adaptive immunity by inducing uric acid and activating inflammatory dendritic cells. J Exp Med 205: 869–882. 18362170 10.1084/jem.20071087 PMC2292225

[29] Li H, Willingham SB, Ting JP, Re F (2008) Cutting edge: Inflammasome activation by alum and alum’s adjuvant effect are mediated by Nlrp3. J Immunol 181: 17–21. 18566365 10.4049/jimmunol.181.1.17 PMC2587213

[30] Schweneker K, Gorka O, Schweneker M, Poeck H, Tschopp J, et al.. (2012) The mycobacterial cord factor adjuvant analogue trehalose-6,6′-dibehenate (TDB) activates the Nlrp3 inflammasome. Immunobiology: DOI 10.1016/j.imbio.2012.07.029. 10.1016/j.imbio.2012.07.029 22921586

[31] Greten FR, Arkan MC, Bollrath J, Hsu LC, Goode J, et al (2007) NF-kappaB is a negative regulator of IL-1beta secretion as revealed by genetic and pharmacological inhibition of IKKbeta. Cell 130: 918–931. 17803913 10.1016/j.cell.2007.07.009 PMC2134986

[32] Kayagaki N, Warming S, Lamkanfi M, Walle LV, Louie S, et al (2011) Non-canonical inflammasome activation targets Caspase-11. Nature 479: 117–121. 22002608 10.1038/nature10558

[33] Dinarello CA (2011) Interleukin-1 in the pathogenesis and treatment of inflammatory diseases. Blood 117: 3720–3732. 21304099 10.1182/blood-2010-07-273417 PMC3083294

[34] Gross O, Yazdi AS, Thomas CJ, Masin M, Heinz LX, et al (2012) Inflammasome activators induce Interleukin-1alpha secretion via distinct pathways with differential requirement for the protease function of Caspase-1. Immunity 36: 388–400. 22444631 10.1016/j.immuni.2012.01.018

[35] Chung Y, Chang SH, Martinez GJ, Yang XO, Nurieva R, et al (2009) Critical regulation of early Th17 cell differentiation by Interleukin-1 signaling. Immunity 30: 576–587. 19362022 10.1016/j.immuni.2009.02.007 PMC2705871

[36] Wells CA, Salvage-Jones JA, Li X, Hitchens K, Butcher S, et al (2008) The macrophage-inducible C-type lectin, mincle, is an essential component of the innate immune response to *candida albicans.* . J Immunol 180: 7404–7413. 18490740 10.4049/jimmunol.180.11.7404

[37] Wenzel J, Held C, Palmisano R, Teufel S, David JP, et al (2011) Measurement of TLR-induced macrophage spreading by automated image analysis: Differential role of MyD88 and MAPK in early and late responses. Front Physiol 2: 71. 22028692 10.3389/fphys.2011.00071 PMC3198511

