# Supplementary figures and images for: The Mincle-Activating Adjuvant TDB Induces MyD88-Dependent Th1 and Th17 Responses through IL-1R Signaling
Source: PLoS One. 2013 Jan 7;8(1):e53531. doi: 10.1371/journal.pone.0053531 (PMC3538599; doi:10.1371/journal.pone.0053531)

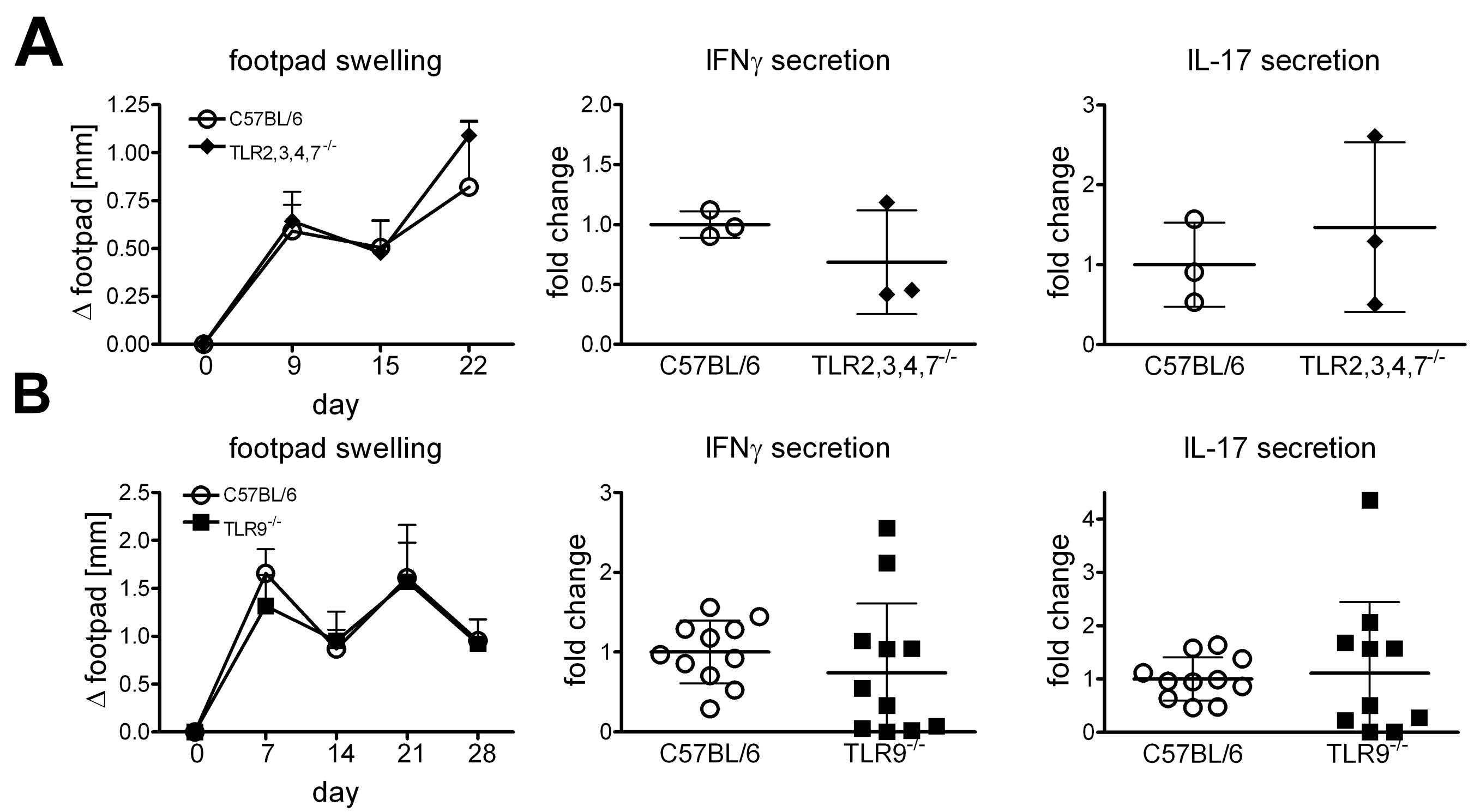

Supplement: Figure S2 — TLR2,3,4,7 and 9 seem dispensable for DDA/TDB adjuvanticity. Footpad swelling, IFNγ and IL-17 secretion in TLR2,3,4,7−/− and C57BL/6 controls (A). Data presented as mean ± SD from 1 experiment with 3 mice/group. Footpad swelling, IFNγ and IL-17 secretion in TLR9−/− and C57BL/6 controls (B). Data presented as mean ± SD from 3 independent experiments with 3–5 mice/group. Cytokine production of cells isolated from the draining lymph nodes. (TIF) [file pone.0053531.s002.tif]

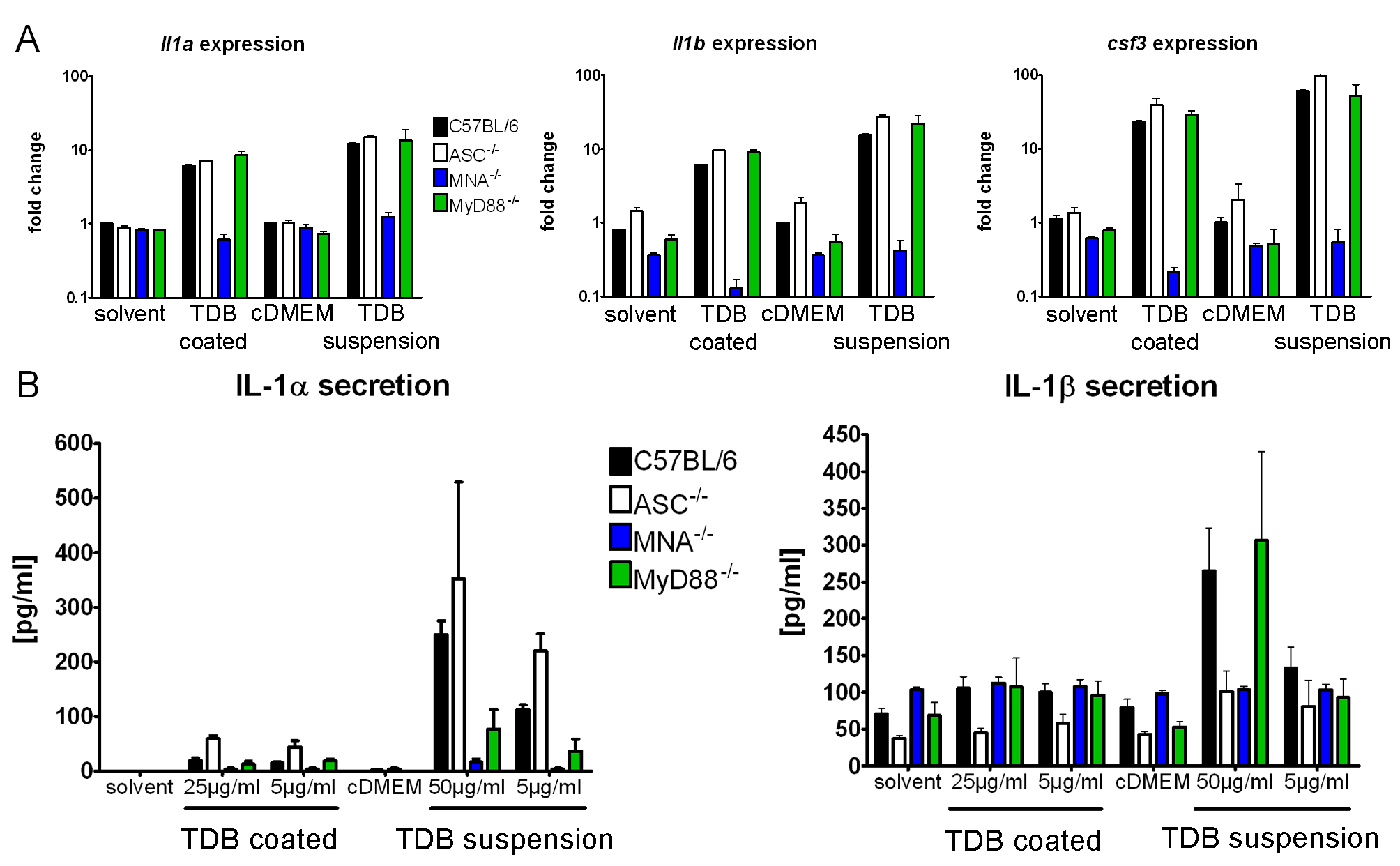

Supplement: Figure S3 — TDB-induced expression and release of IL-1α and IL-1β. Expression of Il1a, Il1b and Csf3 (A), and IL-1α and IL-1β secretion (B). Bone marrow-derived DC were stimulated for 24 h with plate-coated TDB (solvent control isopropanol) or TDB in suspension. 2.5×10^5^ cells/well were seeded for cytokine release (TDB concentration as indicated) and 5×10^5^ cells/well for qRT-PCR (5 µg/ml TDB). Fold change calculated against DC from C57BL/6 mice in medium. One experiment performed in duplicates. (TIF) [file pone.0053531.s003.tif]

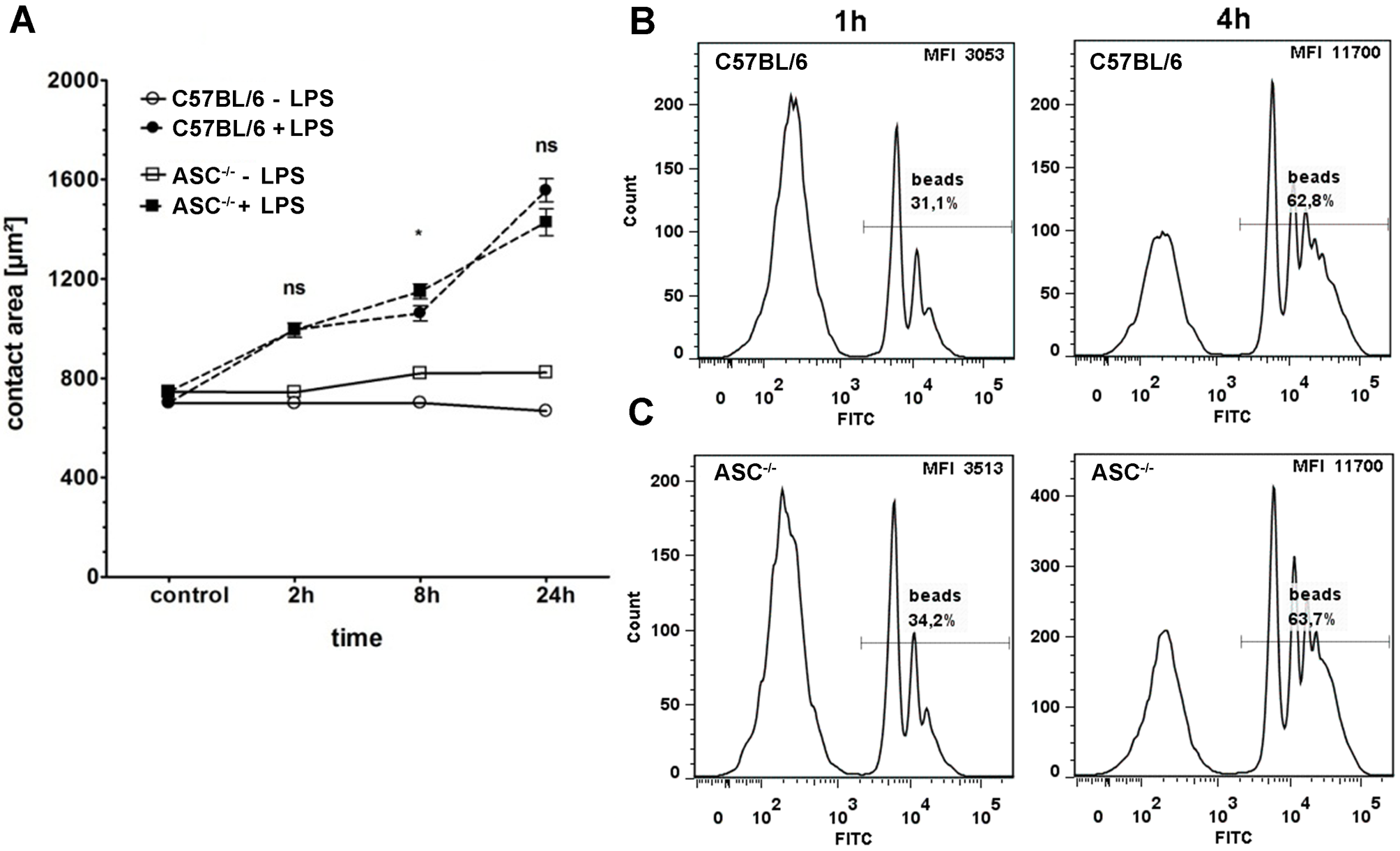

Supplement: Figure S4 — ASC−/− macrophages show no defects in spreading kinetics and phagocytosis capacity. Spreading kinetics (A) of C57BL/6 (circles) and ASC−/− (squares) BMM stimulated with LPS (closed symbols) or media control (open symbols). Mean ± SEM of at least 300 cells per condition. Statistical significance refers to the comparison of LPS stimulated C57BL/6 and ASC−/− BMM. One experiment performed. Phagocytosis capacity of C57BL/6 (B) and ASC−/− (C) BMM. Cells were incubated with fluorescent latex beads (1 µm; MOI 20) for 1 h and 4 h. Percentages of cells which phagocytosed beads determined flow cytometry. One of two representative experiments shown. (TIF) [file pone.0053531.s004.tif]
